# Metabolic syndrome in Russian adults: associated factors and mortality from cardiovascular diseases and all causes

**DOI:** 10.1186/1471-2458-10-582

**Published:** 2010-09-29

**Authors:** Oleg Sidorenkov, Odd Nilssen, Andrej M Grjibovski

**Affiliations:** 1 Institute of Community Medicine, University of Tromsø, Tromsø, Norway; 2 Norwegian Institute of Public Health, Oslo, Norway; 3 International School of Public Health, Northern State Medical University, Arkhangelsk, Russia

## Abstract

**Background:**

Metabolic syndrome (MetS) is a cluster of four major obesity-related risk factors for cardiovascular disease (CVD). Russia has one of the highest CVD mortality in the world, but its association with MetS remains unknown. Also little is known about factors associated with MetS and its components in Russia.

**Methods:**

Data on 3555 adults aged 18-90 years were collected in a cross-sectional study in 2000. MetS was defined by the International Diabetes Federation (IDF) and National Cholesterol Education Program (NCEP) criteria. Sex-specific associations between the IDF-defined MetS, its components, and life-style, socio-economic factors and laboratory indicators, were analysed using multivariable Poisson regression. Vital status of the study participants was identified by July 2009. Sex-specific associations between MetS and stroke, Coronary Heart Disease (CHD), CVD and all-cause death, were studied by Poisson regression adjusted for age, smoking, alcohol and history of CVDs.

**Results:**

After adjustment for all studied factors except BMI, age, serum GGT, C-reactive protein and AST-to-ALT ratio were associated with MetS in both genders. Additionally, MetS was associated with sedentary lifestyle in women and with smoking in men. In the same regression model drinking alcohol 2-4 times a month and consumption of five or more alcohol units at one occasion in men, and drinking alcohol 5 times or more a month in women were inversely associated with MetS. After a 9-year follow-up, MetS was associated with higher risk of death from stroke (RR = 3.76, 95% CI:1.35-10.46) and from either stroke or myocardial infarction (MI, RR = 2.87, 95% CI:1.32-6.23) in men. No associations between MetS and any of the studied causes of death were observed in women.

**Conclusion:**

Factors associated with MetS in both genders were age, GGT, C-reactive protein, and AST-to-ALT ratio. Moderate frequency of alcohol consumption and binge drinking in men and higher leisure time physical activity in women, were inversely associated with MetS.

Positive associations between MetS and mortality were only observed for deaths from stroke and either stroke or MI in men.

## Background

The metabolic syndrome (MetS) is a cluster of four major cardiovascular disease (CVD) risk factors; obesity, insulin resistance (hyperglycemia), arterial hypertension and dyslipidemia where obesity and insulin resistance are the core elements [1]. Other important characteristics of MetS include low-grade inflammation, endothelial dysfunction, plasma hypercoagulability and atherosclerosis [2].

MetS is associated with increased CVD and all-cause mortality [3,4]. Moreover, it may be used as an alternative to the classic coronary heart disease (CHD) risk assessment scale such as the Framingham Risk Score [5]. The prevalence of MetS varies greatly between countries and ethnic groups [6]. Among Europeans and white Americans it varies between 20% and 30% with similar gender distribution [7,8]. Due to its high prevalence, MetS is considered as the major public health problem in Europe, and, particularly in the USA, where obesity and overweight are the second leading cause of preventable death accounting for 300.000 deaths per year [9].

The prevalence of MetS is associated with life-style, demographic, socio-economic, and genetic factors. Age, body mass index, postmenopausal status, diet rich in saturated fats, carbohydrates, and smoking have been positively associated with MetS, while inverse associations have been shown for physical activity, education, income, and alcohol intake [7,10-12].

Cardiovascular mortality in Russia is about four times higher than in Western Europe and the gap is the largest among middle aged men [13]. Although there is evidence for a high contribution of hazardous level of alcohol consumption to high death rates in Russia [14-16], other factors also need to be investigated. As MetS represents a cluster of the four of six major cardiovascular risk factors strongly associated with CVD mortality, one might expect similar high rates of MetS or its components in Russia.

Despite the fact that determinants of MetS and its contribution to mortality in Europe and North America receive much attention by the research community, it remains one of the least studied factors in Russia. In an earlier study we showed that while the prevalence of MetS among Russian women in 2000 was comparable with findings from other European countries, among men it was a half of that [17].

The aim of this study was to further explore the data collected in 2000 by studying socio-demographic and lifestyle correlates of MetS and associations between the MetS and CVD-and all-cause mortality after 9 years of follow-up.

## Methods

### Study sample

The data were obtained from a cross-sectional population-based study conducted in 2000 in Arkhangelsk, Northwest Russia. Detailed information on study design, sampling procedure and data collection is presented elsewhere [17-19]. In brief, we invited 3745 subjects aged ≥18 years from the patient register at the Semashko outpatient clinic in Arkhangelsk. Most of the participants were consecutively recruited when they came for their obligatory annual medical examinations. Others, particularly pensioners from the area served by the Semashko clinic, were specifically invited to participate in this study. Only 40 individuals refused to participate (response rate 98.9%).

### Data collection

Data on education, occupational status, use of medications, history of myocardial infarction (MI), diabetes mellitus, and stroke as well as typical patterns of leisure time physical activity, smoking, frequency and amount of alcohol consumption, frequency of fresh fruits and vegetables intake with no specified time-frames were collected using a 6-page comprehensive questionnaire. Blood pressure (BP) was measured three times. The average of the two last readings was used in the study. Waist circumference (WC) was measured at the umbilical level. Weight and height were measured with subjects in light clothing and without shoes. Venous blood samples were drawn and centrifuged within 15-25 min. Most of the participants fasted prior to testing.

### Laboratory analyses

Enzymatic colorimetric tests were used to measure TC (cholesterol esterase, cholesterol oxidase) and TG (lipoprotein lipase, glycerokinase, and glycerophosphate oxidase). HDL-C was measured by a homogenous enzymatic colorimetric test (PEG cholesterol esterase, and PEG peroxidase). All biochemical analyses of serum lipids were performed using a Hitachi 737 analyzer. Gamma-glutamyltransferase (GGT) was measured by an enzymatic colorimetric test (standardized method, Roche). Aspartate-aminotransferase (AST) and alanin-aminotransferase (ALT) were measured photometrically by Hitachi 917 analyzer. Serum C-reactive protein (CRP) was measured by particle-enhanced immunoturbidimetric assay in a Roche Modular P analyzer (Roche Diagnostics GmbH, D-68298 Mannheim). Glycated hemoglobin (HBA1c), which reflects the mean glucose level over the preceding 2-3 months, was assessed by Bio-Rad Variant II HPLC system with reagents from Bio-Rad Laboratories (Inc., Hercules, CA 94547, USA).

The inter-assay and intra-assay coefficients of variation for all laboratory tests were under 3%.

### Definition of the metabolic syndrome

MetS was defined according to the International Diabetes Federation (IDF) [6] and National Cholesterol Education Program (NCEP) [20]criteria. We applied cut-offs for WC as it was recommended for europids (men ≥94 cm, women ≥80 cm) and (men ≥102 cm, women ≥88 cm), respectively, for IDF and NCEP. We used HBA1c as the measurement of hyperglycemia (defined as HBA1c ≥6.1%, and/or self-reported diabetes, and/or receiving treatment for diabetes).

### Description of the variables

Education was divided into 3 categories: low (primary or secondary school), average (vocational school or incomplete university education) and high (complete university education). As income level was difficult to determine due to high inflation and collapsing economy due to the crisis and default in 1998-99, we used data on self-reported occupational status as a "surrogate" measure for income. Income level was defined according to official data on average salary levels in different sectors of the economy for year 2000 [21]. Five categories were generated: very low, low, medium, high and unknown. Occasional and daily smokers comprised the group of smokers, while non-smokers and ex-smokers comprised the non-smoking group. Leisure-time physical activity was dichotomized as "inactive" or "sedentary lifestyle" (predominantly sitting activity like reading, watching TV) and "active" (walking or bicycling or yard working at least 4 hours per week, regular training and professional sport). Intake of fresh fruits or vegetables was dichotomized as "low" (once a week or less) and "high" (2 times a week or more). Alcohol consumption was presented by two variables: frequency of alcohol intake, and number of alcohol units (AU) consumed on one occasion. One AU was equal to 13.8 g of pure alcohol. The frequency of alcohol consumption was divided into 4 groups: abstainers, ≤1 times a month, 2-4 times a month, ≥5 times a month. The number of AU consumed at one drinking session was divided into 3 categories: abstainers, 1-4 AU and ≥5 AU (later referred to as binge drinking). Normal weight, overweight and obesity were defined as a BMI <25, 25-29.9, and ≥30 kg/m^2^, respectively. As the distribution of the liver enzymes and CRP was right-skewed, we used logarithmically transformed values in the regression models.

Altogether 150 individuals had missing data on one or more variables and were excluded from the analyses. The final sample consisted of 3555 individuals (1918 men and 1637 women) aged 18-90 years or 96% of the initial sample.

### Statistical analyses

Differences in the distribution of the studied characteristics between genders were studied by Pearson's chi-squared tests and unpaired t-tests for categorical and continuous data, respectively. Gender-specific associations between MetS defined by the IDF criteria, its individual components and socio-demographic, lifestyle and metabolic factors were calculated using Poisson regression with robust variance estimates as recommended by Barros and Hirakata [22], and are presented as crude and adjusted prevalence ratios (PR) with 95% confidence intervals (CI).

### Follow-up study

In July 2009 we collected data on the vital status of all study participants, using the mortality register of the Arkhangelsk Regional Healthcare Department which is based on data from medical death certificates. Causes of death were coded using the International Classification of Diseases, 10th Revision (ICD-10). The study end-points were: death from coronary heart disease (CHD) (I20-25); death from stroke (I60-64); death from either myocardial infarction (MI) or stroke (I21-23; I60-64); CVD death (I00-99); and all-cause death. By July 2009, 200 subjects of the 3555 participants had died and in 97 of the cases (48%) the diagnosis was verified by autopsy. To study associations between MetS in 2000 and mortality by 2009, we used both IDF and NCEP definitions of MetS to increase comparability of the findings with other studies. Gender-specific risk ratios (RR) were calculated by Poisson regression.

All analyses were performed using STATA 10 (STATA Corp, TX, USA). The study was approved by the Regional Ethical Committee in Norway.

## Results

### Sample characteristics

Participants' background characteristics and the prevalence of MetS are presented in Table 1. Men were younger, had higher income, but lower education than women. They were more physically active, smoked more, drank alcohol more often and had higher levels of alcohol intake at one drinking session. About 50% of the men reported binge drinking, by contrast to 15% among women. Vodka/liquor constituted about 66% and 45% of the total consumption in men and women, respectively (data not shown). Men also had higher levels of liver enzymes and CRP. The prevalence of MetS in men was half of that in women (Table 1).

**Table 1:** Prevalence of the metabolic syndrome stratified by gender, age, BMI, laboratory tests, socio-demographic and the life-style characteristics

Socio-demographic and the life-style characteristics	Men		Women		**P-value**^**2**^
		
	N (%)	**MetS, % with (95% CI)**^**1**^	N (%)	**MetS, % with (95% CI)**^**1**^	
Age, years					0.002
18-29	515 (26.9)	1.75 (0.9-3.4)	347 (21.2)	3.8 (2.1-6.5)	
30-39	352 (18.4)	6.25 (4.1-9.5)	303 (18.5)	8.6 (5.8-12.5)	
40-49	441 (23.0)	11.3 (8.6-14.8)	400 (24.4)	22.8 (18.8-27.2)	
50-59	298 (15.5)	14.8 (11.0-19.4)	290 (17.7)	41.4 (35.7-47.3)	
60+	312 (16.3)	18.3 (14.2-23.1)	297 (18.1)	45.5 (39.7-51.3)	
BMI, kg/m^2^					< 0.001
< 25.0	989 (51.5)	0.3 (0.1-1.0)	781 (47.7)	4.0 (2.8-5.7)	
25.0-29.9	707 (36.9)	9.3 (7.4-11.8)	515 (31.5)	28.9 (25.1-33.1)	
≥30.0	222 (11.6)	50.9 (44.1-57.6)	341 (20.8)	60.1 (54.7-65.3)	
Education					< 0.001
Secondary school	435 (22.7)	10.3 (7.7-13.7)	426 (26.0)	31.9 (27.6-36.6)	
College	1170(61.0)	7.9 (6.4-9.6)	774 (47.3)	21.3 (18.5-24.4)	
University	313 (16.3)	14.4 (10.8-18.9)	437 (26.7)	19.2 (15.7-23.3)	
Income					< 0.001
Very low	283 (14.8)	17.0 (12.9-22.0)	379 (23.2)	43.8 (38.8-49.0)	
Low	136 (7.1)	14.7 (9.4-22.0)	740 (45.2)	20 (17.2-23.1)	
Medium	144 (7.5)	6.9 (3.6-12.7)	189 (11.5)	23.8 (18.1-30.7)	
High	1058(55.2)	9.3 (7.6-11.2)	34 (2.1)	17.7 (7.4-35.2)	
Unknown	297 (15.5)	2.0 (0.8-4.6)	295 (18.0)	6.8 (4.3-10.4)	
Sedentary lifestyle					< 0.001
Yes	437 (22.8)	14.0 (10.9-17.7)	656 (40.1)	32.2 (28.6-35.9)	
No	1481(77.2)	8.2 (6.9-9.7)	981 (59.9)	17.7 (15.4-20.3)	
Current smoking					< 0.001
Yes	1085(56.6)	7.5 (6.0-9.2)	348 (21.3)	13.2 (9.9-17.3)	
No	833 (43.4)	12.1 (10.0-14.6)	1289(78.7)	26.3 (23.9.28.8)	
Low fresh fruits/vegetables intake					0.01
Yes	779 (40.6)	8.2 (6.4-10.4)	599(36.6)	25.5 (22.1-29.3)	
No	1139(59.4)	10.4 (8.7-12.3)	1038(63.4)	22.4 (19.9-25.0)	
Frequency of alcohol intake					< 0.001
Abstainers	230 (12.0)	15.2 (11.0-20.7)	445 (27.2)	34.2 (29.8-38.8)	
≤ 1 times a month	434 (22.6)	12.4 (9.6-16.0)	542 (33.1)	25.3 (21.7-29.2)	
2-4 times a month	979 (51.0)	7.2 (5.7-9.0)	571 (34.9)	15.9 (13.1-19.3)	
≥5 times a month	275 (14.3)	8.4 (5.5-12.4)	79 (4.8)	6.3 (2.4-14.8)	
Number of AU on occasion					< 0.001
Abstainers	230 (12.0)	15.2 (11.0-20.7)	445 (27.2)	34.2 (29.8-38.8)	
1-4 AU	780 (40.5)	9.5 (7.6-11.8)	946 (57.7)	20.0 (17.5-22.7)	
≥ 5 AU	912 (47.5)	8.0 (6.4-10.0)	248 (15.1)	17.7 (13.3-23.2)	
Self-reported MI or stroke	56 (2.9)		51 (3.1)		0.768
GGT, U/l, mean (SD)	43.7 (60.8)		28.4 (39.8)		< 0.001
AST, U/l, mean (SD)	29.5 (22.7)		23.6 (13.7)		< 0.001
ALT, U/l, mean (SD)	20.7 (20.8)		12.9 (12.8)		< 0.001
AST/ALT, mean (SD)	1.8 (0.9)		2.1 (0.8)		< 0.001
CRP, mg/l, mean (SD)	3.2 (9.1)		2.6 (5.6)		0.02

Metabolic syndrome^3^	182/1918	9.5 (8.2-10.9)	385/1637	23.5 (21.5-25.7)	< 0.001

^1 ^95% CI for proportions calculated using Wilson procedure

^2 ^p-values for the differences between genders

^3 ^Metabolic syndrome defined according to the modified IDF criteria

### Correlates of the metabolic syndrome

Among men, MetS was positively associated with age, BMI, sedentary lifestyle, GGT and CRP; and inversely associated with income, smoking, frequency and amount of alcohol intake as well as the AST-to-ALT ratio in the crude analysis. After adjustment for all studied factors except BMI, the associations between MetS and income disappeared. Additional adjustment for BMI attenuated most of the associations except the positive association with age, and inverse associations with the AST-to-ALT ratio, frequency and amount of alcohol consumption (Table 2).

**Table 2:** Sex-specific crude and multivariate adjusted PRs for metabolic syndrome¹

Factor	Men			Women		
	
	**Model 1**^**2**^	Model 2	Model 3	**Model 1**^**2**^	Model 2	Model 3
Age						
18-29	Reference	Reference	Reference	Reference	Reference	Reference
30-39	3.58 (1.67-7.68)	2.24 (0.82-6.18)	1.4 (0.57-3.43)	2.29 (1.20-4.38)	1.55 (0.80-2.98)	1.42 (0.78-2.58)
40-49	6.49 (3.23-13.04)	3.75 (1.44-9.78)	2.03 (0.88-4.68)	6.07(3.46-10.67)	3.43 (1.90-6.19)	2.50 (1.45-4.33)
50-59	8.45 (4.18-17.06)	4.98 (1.89-13.14)	2.91 (1.25-6.75)	11.1(6.37-19.16)	5.39 (2.93-9.89)	3.76 (2.12-6.67)
60+	10.45 (5.25-20.82)	6.58 (2.34-18.49)	5.06 (2.09-12.21)	12.1(7.02-20.98)	5.09 (2.69-9.65)	3.97 (2.17-7.26)
P for trend	< 0.001	< 0.001	< 0.001	< 0.001	< 0.001	< 0.001
Education						
Secondary school	Reference	Reference	Reference	Reference	Reference	Reference
College	0.76 (0.54-1.07)	1.03 (0.73-1.47)	1.23 (0.86-1.76)	0.67 (0.55-0.81)	0.96 (0.78-1.18)	0.89 (0.73-1.09)
University	1.39 (0.94-2.05)	1.10 (0.73-1.65)	1.18 (0.79-1.77)	0.60 (0.48-0.76)	0.80 (0.62-1.03)	0.89 (0.69-1.13)
P for trend	0.190	0.660	0.375	< 0.001	0.085	0.329
Income						
Very low	1.15 (0.71-1.86)	0.87 (0.48-1.59)	0.81 (0.46-1.44)	2.19 (1.82-2.63)	0.99 (0.77-1.26)	0.97 (0.76-1.23)
Low	Reference	Reference	Reference	Reference	Reference	Reference
Medium	0.47 (0.23-0.97)	0.67 (0.32-1.40)	0.62 (0.28-1.33)	1.19 (0.89-1.60)	1.11 (0.82-1.50)	1.03 (0.75-1.41)
High	0.62 (0.40-0.98)	0.84 (0.52-1.33)	0.81 (0.50-1.31)	0.88 (0.42-1.85)	0.97 (0.47-1.98)	1.01 (0.53-1.92)
Unknown	0.14 (0.06-0.33)	0.83 (0.26-2.62)	1.22 (0.45-3.31)	0.34 (0.22-0.53)	0.69 (0.45-1.07)	0.79 (0.52-1.19)
Fresh fruits/vegetab. intake; high vs. low	1.26 (0.94-1.69)	1.21 (0.90-1.63)	1.17(0.85-1.58)	0.88 (0.73-1.05)	1.05 (0.88-1.25)	1.06 (0.90-1.25)
Current smoking	0.62 (0.47-0.81)	0.74 (0.56-0.97)	1.08 (0.81-1.45)	0.50 (0.38-0.67)	0.98 (0.74-1.30)	1.04 (0.80-1.35)
Sedentary lifestyle	1.7 (1.28-2.28)	1.33 (0.99-1.81)	1.13 (0.84-1.52)	1.81 (1.52-2.16)	1.31 (1.11-1.55)	1.19 (1.01-1.40)
Frequency of alcohol intake						
Abstainers	Reference	Reference	Reference	Reference	Reference	Reference
≤1 times a month	0.82 (0.55-1.21)	0.90 (0.60-1.35)	0.66 (0.45-0.98)	0.74 (0.61-0.90)	1.13 (0.92-1.39)	1.04 (0.85-1.27)
2-4 times a month	0.47 (0.32-0.67)	0.62 (0.42-0.93)	0.59 (0.41-0.85)	0.47 (0.37-0.59)	0.96 (0.75-1.22)	0.90 (0.71-1.14)
≥5 times a month	0.55 (0.33-0.90)	0.76 (0.46-1.26)	0.61 (0.37-1.00)	0.19 (0.08-0.44)	0.42 (0.19-0.97)	0.58 (0.26-1.30)
P for trend	< 0.001	0.045	0.030	< 0.001	0.202	0.190
Body Mass Index						
< 25	Reference	Reference	Reference	Reference	Reference	Reference
25.0-29.9	30.78 (9.71-97.51)	-	22.0 (6.61-73.24)	7.3 (5.0-10.6)	-	4.4 (3.0-6.5)
30.0-34.9	163.1(52.2-509.6)	-	105.0 (31.6-349.1)	14.5 (10.1-20.9)	-	7.0 (4.6-10.5)
≥35	195.7 (61.0-628.1)	-	132.9 (39.1-451.5)	16.8 (11.5-24.5)	-	7.3 (4.8-11.3)
Number of AU on one occasion^3^						
Abstainers	Reference	Reference	Reference	Reference	Reference	Reference
1-4 AU	0.61 (0.42-0.89)	0.78 (0.53-1.15)	0.69 (0.48-0.99)	0.58 (0.49-0.70)	1.04 (0.85-1.26)	0.96 (0.79-1.17)
≥ 5 AU	0.52 (0.36-0.75)	0.61 (0.40-0.93)	0.52 (0.35-0.76)	0.52 (0.38-0.70)	1.07 (0.79-1.46)	1.06 (0.78-1.43)
P for trend	0.003	0.017	0.001	< 0.001	0.631	0.893
Log GGT	4.3 (3.18-5.81)	1.83 (1.22-2.75)	1.28 (0.82-2.0)	4.08 (3.28-5.07)	1.69 (1.27-2.26)	1.62 (1.22-2.15)
Log AST/Log ALT	0.11 (0.05-0.27)	0.09 (0.04-0.22)	0.19 (0.08-0.46)	0.34 (0.21-0.56)	0.56 (0.33-0.94)	0.82 (0.51-1.32)
Log CRP	2.28 (1.9-2.73)	1.63 (1.28-2.09)	1.27 (0.93-1.72)	3.16 (2.73-3.65)	2.17 (1.82-2.59)	1.61 (1.09-1.12)

^1 ^Metabolic syndrome is defined according to modified IDF criteria

^2 ^Model 1: crude PRs. Model 2 estimates for the MetS adjusted for age, education, income, frequency of fresh fruit and vegetable intake, smoking habits, physical activity and the frequency of alcohol consumption. Model 3: estimates for the MetS adjusted for all covariates in Model 2 plus BMI

^3 ^The PRs for "Number of AU on one occasion" in Model 2 and Model 3 adjusted as before excluding variable "Frequency of alcohol consumption"

In women, MetS was positively associated with BMI, age, very low income, sedentary lifestyle, GGT and CRP, and inversely associated with education, unknown income category, smoking, frequency and amount of alcohol consumption as well as AST-to-ALT ratio in crude analysis. After adjustment for all study factors except BMI, the associations between MetS and income, education, smoking and alcohol disappeared. After further adjustment for BMI, only age, sedentary lifestyle, GGT and CRP remained associated with MetS.

### Correlates of the individual metabolic components

In the multivariable analysis of the MetS components (Table 3), frequency and volume of alcohol intake were inversely associated with prevalence of hypertriglyceridemia (high-TG), low levels of high density lipoproteins (low-HDL-C) and hyperglycemia in men. Similar results were found for frequency of alcohol consumption in women. The volume of consumed alcohol increased HDL-C levels, but showed weaker association in women than in men. Current smoking in men and sedentary lifestyle in women, were related to unfavorable lipid status. It was associated with 40% lower rates of central adiposity in men, as compared to non-smokers.

**Table 3:** Gender-specific multivariable adjusted PRs¹ for individual components of the metabolic syndrome defined according to modified IDF criteria by frequency and volume of alcohol consumption, other life-style factors, levels of GGT, AST-to-ALT ratio and C-reactive protein

	Metabolic abnormalities				
	
	High TG	Low HDL-C	**Central obesity**^**2**^	Hypertension	Hyperglycemia
Men (1918)					
Frequency of alcohol intake					
Never	Reference	Reference	Reference	Reference	Reference
1 time a month	0.85(0.65-1.12)	0.94(0.75-1.18)	1.11(0.83-1.49)	1.01(0.90-1.14)	0.52(0.26-1.02)
2-4 times a month	0.76(0.59-0.97)	0.80(0.65-0.98)	0.95(0.71-1.26)	0.96(0.86-1.08)	0.59(0.33-1.05)
≥5 times a month	1.02(0.76-1.36)	0.85(0.64-1.12)	0.89(0.62-1.29)	0.97(0.83-1.13)	0.42(0.13-1.37)
P for trend	0.743	0.059	0.243	0.410	0.090
Number of AU on one occasion^3^					
Abstainers	Reference	Reference	Reference	Reference	Reference
1-4 AU	0.90(0.71-1.16)	0.86(0.70-1.06)	1.00(0.76-1.33)	0.97(0.87-1.09)	0.56(0.30-1.02)
≥5 AU	0.73(0.57-0.95)	0.79(0.63-0.98)	0.96(0.71-1.29)	0.96(0.86-1.08)	0.50(0.27-0.95)
P for trend	0.005	0.034	0.682	0.603	0.053
Current smoking	1.12(0.95-1.33)	1.25(1.07-1.46)	0.61(0.50-0.74)	0.95(0.87-1.03)	0.87(0.52-1.46)
Sedentary lifestyle	0.98(0.82-1.19)	1.11(0.93-1.31)	1.17(0.96-1.44)	1.04(0.95-1.15)	1.14(0.69-1.90)
LogAST-to-LogALT	0.39(0.22-0.67)	0.90(0.62-1.32)	0.19(0.11-0.33)	1.14(0.95-1.37)	0.82(0.22-3.04)
Log GGT	2.32(1.80-2.98)	0.80(0.59-1.08)	1.78(1.32-2.40)	1.21(1.05-1.40)	1.45(0.68-3.07)
Log CRP	0.92(0.76-1.11)	1.52(1.33-1.75)	1.57(1.30-1.89)	1.00(0.91-1.10)	1.51(0.95-2.41)
Women (1637)					
Frequency of alcohol intake					
Never	Reference	Reference	Reference	Reference	Reference
1 time a month	0.96(0.76-1.22)	0.90(0.78-1.04)	1.12(1.00-1.26)	0.96(0.85-1.09)	1.0(0.57-1.75)
2-4 times a month	0.83(0.63-1.10)	0.75(0.64-0.88)	1.03(0.91-1.18)	0.96(0.82-1.12)	0.42(0.17-1.04)
≥5 times a month	0.52(0.25-1.09)	0.48(0.32-0.72)	0.75(0.52-1.08)	0.76(0.45-1.30)	-
P for trend	0.065	< 0.001	0.605	0.346	0.030
Number of AU on one occasion^3^					
Abstainers	Reference	Reference	Reference	Reference	Reference
1-4 AU	0.88(0.71.1.11)	0.82(0.71-0.94)	1.06(0.95-1.18)	0.94(0.84-1.06)	0.77(0.44-1.35)
≥5 AU	0.98(0.70-1.37)	0.84(0.69-1.02)	1.15(0.98-1.36)	1.03(0.84-1.27)	0.79(0.28-2.20)
P for trend	0.684	0.039	0.087	0.915	0.442
Current smoking	1.40(1.08-1.82)	1.16(1.0-1.35)	0.94(0.81-1.09)	0.87(0.71-1.08)	1.08(0.37-3.18)
Sedentary lifestyle	1.24(1.03-1.50)	1.11(0.99-1.25)	1.06(0.97-1.16)	0.96(0.87-1.06)	0.98(0.60-1.60)
LogAST-to-LogALT	0.55(0.31-0.99)	1.17(0.88-1.55)	0.51(0.37-0.68)	0.92(0.70-1.21)	0.78(0.21-2.96)
Log GGT	1.93(1.42-2.62)	1.25(1.01-1.55)	1.27(1.08-1.50)	1.19(0.98-1.44)	2.18(0.99-4.83)
Log CRP	1.49(1.21-1.82)	1.42(1.25-1.61)	1.68(1.51-1.86)	1.29(1.14-1.45)	1.98(1.27-3.07)

^1 ^The regression models are adjusted for: age, intake of fresh fruits or vegetables, level of leisure time physical activity, income, education, smoking, frequency of alcohol intake and body mass index (BMI)

^2 ^PRs for central obesity are given for the regression model excluding BMI

^3 ^PRs for "Number of alcohol units (AU) on one occasion" are given for the regression model excluding "Frequency of alcohol intake"

Serum levels of GGT and CRP in women were positively related to all five metabolic components (Table 3). High serum levels of GGT were the strongest metabolic marker in men, in whom it was related to increased prevalence of hypertriglyceridemia, central obesity and hypertension. The AST-to-ALT ratio was inversely associated with hypertriglyceridemia and central obesity in both genders, although the strength of association was larger in men.

### Metabolic syndrome and mortality

MetS as defined by the IDF criteria was associated with more than 6 times higher risk of death from stroke among men after 9 years of observation in (Table 4, Model 1). Adjustment for age, history of CVD, smoking and alcohol attenuated the association, but the risk of death from stroke was still more than 3 times higher for men with MetS. Death from either stroke or myocardial infarction occurred twice as common among the men with IDF-defined MetS (Model 3). In women, the association between MetS and death from the former causes was much less pronounced (Model 1) and disappeared after adjustment for other covariates.

**Table 4:** Risk ratios (RR) with 95% confidence intervals (CI) for death from CHD, stroke, myocardial infarction or stroke, CVD and all causes associated with metabolic syndrome during the 9-year follow-up

	RR (95% CI)			
	
**Models**^**1**^	Men (1918)		Women (1637)	
	
	IDF	NCEP	IDF	NCEP
**N (%)**	**182 (9.5)**	**191 (10.0)**	**385 (23.5)**	**343 (21.0)**

CHD death, N	44		18	
Model 1	1.84(0.81-4.18)	1.74(0.76-3.95)	1.64(0.61-4.39)	3.07(1.20-7.83)
Model 2	0.97(0.41-2.26)	0.87(0.37-2.03)	0.99(0.34-2.94)	1.53(0.54-4.34)
Model 3	0.78(0.32-1.91)	0.73(0.30-1.76)	0.86(0.27-2.67)	1.45(0.49-4.33)

Stroke death, N	15		17	
Model 1	6.36(2.29-17.67)	7.91(2.90-21.58)	1.77(0.66-4.77)	2.64(1.01-6.89)
Model 2	3.32(1.25-8.83)	4.07(1.55-10.72)	0.95(0.36-2.53)	1.23(0.48-3.14)
Model 3	3.16(1.11-9.00)	3.76(1.35-10.46)	0.92(0.36-2.33)	1.18(0.48-2.90)

Stroke/MI death, N	25		23	
Model 1	4.49 (1.96-10.26)	6.03 (2.75-13.23)	1.15 (0.46-2.89)	2.01 (0.86-4.71)
Model 2	2.40 (1.09-5.31)	3.12 (1.45-6.73)	0.63 (0.25-1.56)	0.92 (0.40-2.11)
Model 3	2.22 (1.02-4.94)	2.87 (1.32-6.23)	0.60 (0.25-1.43)	0.89 (0.40-1.97)

CVD death, N	66		42	
Model 1	2.34(1.30-4.21)	2.66(1.53-4.64)	2.00(1.09-3.69)	3.43(1.89-6.21)
Model 2	1.25(0.73-2.15)	1.38(0.83-2.28)	1.11(0.63-1.96)	1.58(0.91-2.73)
Model 3	1.08(0.64-1.82)	1.23(0.76-2.00)	1.09(0.63-1.89)	1.54(0.91-2.61)

All-cause death, N	124		76	
Model 1	1.41(0.86-2.34)	1.95(1.26-3.02)	2.12(1.36-3.31)	2.90(1.87-4.49)
Model 2	0.80(0.51-1.27)	1.07(0.71-1.59)	1.15(0.76-1.72)	1.40(0.94-2.09)
Model 3	0.76(0.48-1.18)	1.01(0.69-1.49)	1.13(0.76-1.68)	1.38(0.94-2.04)

^1^Model 1 presents crude estimates

Model 2 presents data adjusted for age.

Model 3 presents data adjusted for age, history of cardiovascular diseases, smoking status and alcohol intake (number of AU taken on one occasion)

The risk of cardiovascular death was almost 2.5 times higher both in men and women with MetS (Table 4, Model 1). However, in both genders these associations disappeared after adjustment for age, and reduced even further after adjustment for other factors. Similar associations were found between all-cause death and MetS in crude analysis, but were attenuated after adjustment for age. The adjusted risk ratio for women was about 40% higher, but did not reach the level of statistical significance (Model 3). No consistent associations between CHD death and MetS were found. Associations between mortality and MetS defined by the NCEP criteria were in the same direction.

## Discussion

To the best of our knowledge this is the first study in Russia on determinants of MetS and its association with all-cause and cardiovascular mortality. The main findings suggest that frequency of alcohol consumption and amount of alcohol consumed at one drinking episode are important correlates of MetS in Northwest Russia. Age, sedentary lifestyle and liver enzymes were also associated with MetS independently of all other studied factors. Moreover, MetS was associated with increased risk of death from stroke and either stroke or myocardial infarction among men during the 9-year observation period. The study discloses sex-specific adjusted relationships between frequency and volume of alcohol consumption in Russia (where these factors are considered to be very important correlates [15,16,23] of cardiovascular death) and all other major cardiovascular risk factors (except smoking) taken both individually and in frames of the MetS concept. GGT, AST, ALT and, particularly, C-reactive protein and AST-to-ALT ratio were associated with MetS and its individual components as expected from the current knowledge[2,24-27].

However, the results should be interpreted cautiously taking into account several limitations of the study. Unemployed and marginalized subjects are likely to be underrepresented. There were 150 participants with missing data on one or several characteristics, although, they did not differ systematically from those included in the analyses by characteristics for which the data were available. Application of modified IDF and NCEP criteria where we used HBA1c serum levels instead of plasma glucose could result in some underestimation of the prevalence of MetS, since the HBA1c is less sensitive. Other limitations related to study design including glycemia measurement have been discussed in details elsewhere [17-19]. Given that diabetes is a risk factor for CVD, the sample was re-analyzed without those who reported diabetes in 2000, but the results were virtually identical.

During the 9-year follow-up we were not able to differentiate those who died in other regions and those who migrated, but did not die. This problem can be attributed to virtually all large Russian longitudinal studies, since there is no national population and mortality registers available for medical research. As a result, the participants, who moved from the Arkhangelsk region during the period of observation, could not be traced and those who died outside the region couldn't be registered. The approximate estimates of loss due to migration during the 9-year period is estimated to be between 15 and 17.5% [28]. Young people (≤30 years) were more likely to migrate to other regions, presumably looking for better work or education. Cardiovascular mortality in this age-group was lowest, compared to the other age-groups, and deaths from external causes accounted for more than 75%. Therefore, it seems unlikely that this loss to follow-up strikingly affected the observed associations between MetS and CVD mortality.

Both frequency of alcohol drinking and amount of alcohol consumed on one occasion were inversely associated with MetS, particularly among men. Interestingly, the crude association between the amount of alcohol consumed on one occasion and MetS was similar for men and women. However, after adjustment, the association persisted only among men. This association seems to be mediated by favorable changes in the lipid profile, but also by improvements in insulin action and lower risk for hyperglycemia (Table 3). A consumption of five or more AU on occasion (about ≥75 g of ethanol) was independently related to 50% lower prevalence of MetS (Table 2, Model 3), and, respectively, 25, 20 and 50% lower prevalence of hypertriglyceridemia, low-HDL-C levels and hyperglycemia (Table 3) among men.

Moderate alcohol consumption is known to increase serum triglycerides level, mainly because of alcohol-stimulated lipolysis [29]. There is evidence that a large part of this TG increase is mediated by contemporary fat consumption [30]. In western communities alcohol intake is often moderate and followed by affluent ingestion of foods, rich in polyunsaturated fats, whereas Russian men still widely combine a pattern of vodka binge drinking with low food intake [15,31]. These cultural peculiarities may explain the decrease of the TG level in response to higher amounts of alcohol consumed among Russian men. Taking into account that more than 50% of men in the study sample reported that they drink at least 5 AU (about one 200 ml glass of vodka) on occasion, and two thirds reported intake of mainly vodka at least two times a month (much the same findings were reported in other studies [32,33] from Russia), we consider that the life-style associated with such a pattern of alcohol intake plays an important role in metabolic risk reduction among Russian men. Thus, gender-specific pattern of alcohol intake and the type of alcohol consumed (high single occasion consumption of strong alcohol by men) together with a confirmed effect of alcohol on serum lipids and insulin resistance might at least partly explain lower rates of MetS among men. It is also possible that this mechanism might also explain the lower metabolic risk in Russian men compared to their Western counterparts. Higher metabolic risk among subjects who abstained from alcohol relative to moderate drinkers, has also been described in longitudinal [34] and cross-sectional [10,35,36] studies. Our results are in line with these findings, suggesting that the pattern of alcohol consumption we found, improves the lipid spectrum by increasing the HDL-C concentration and lowering the low-density lipoprotein cholesterol (LDL-C). Similar results were obtained in another study from Russia where the levels of HDL-C and LDL-C were, respectively, directly and inversely associated with alcohol consumption[37]. Our finding that a consumption of ≥5 AU on occasion is associated with improved glycemic profile, possibly, due to reduction of the insulin resistance, agrees also well with the existing knowledge [29,38].

Low education and low income has been consistently associated with MetS in the US [7,34]. In our study we found no clear effect of these factors. In crude analysis we found slightly lower prevalence of MetS in women with university education and in men with high income. This association disappeared after adjustment. This discrepancy with the findings from other countries may be due to the fact that in Russia the distribution of health outcomes is less strongly linked to socio-economic status compared to the US or the UK. Higher education in Russia does not guarantee high socio-economic status. Moreover, subjects included in the high income category were relatively poor by international standards with an average monthly salary of about 500 USD.

Several studies have reported sedentary life-style as a risk factor for MetS [7,11,12]. We also observed that low leisure-time physical activity was associated with higher prevalence of MetS in both genders, independently of other studied factors.

We observed an inverse association between smoking and MetS (crude analysis), but this association disappeared after adjustment. However, smoking was positively associated with dyslipidemia in both genders and inversely with central adiposity in men. These findings are consistent with previous research suggesting that nicotine increases the energy expenditure, reduces the appetite and stimulates the lipolysis, thus decreasing the risk for obesity [39]. On the other hand, smoking negatively affects the coronary heart system through elevation of blood pressure and development of arteriosclerosis.

Increased serum levels of GGT, CRP and low AST-to-ALT ratio turned out to be independently associated with high metabolic risk, similarly to what has been observed in previous research[2,24-26]. The association of GGT and CRP with MetS was stronger in women, whereas the effect of AST-to-ALT ratio was more pronounced in men (Model 3, Table 2). Several studies have reported that both GGT and CRP synergistically increase with the risk of both metabolic syndrome and obesity as well as with a high alcohol intake [24,25,40]. The pattern of association of the AST-to-ALT ratio is totally different; the ratio tends to be lower (often ≤1) in obese and subjects with the MetS, and higher (often ≥2) in those with high alcohol consumption [27]. One possible explanation of this gender difference is that the association of BMI with MetS in men was much stronger. Another explanation is that the adjusted effects of AST-to-ALT ratio constitute a proxy of the protective action of alcohol which was not fully reflected by self-report [19]. This effect is not evident for GGT and CRP since they are synergistically related to both MetS and alcohol consumption, but it is apparent for the AST-to-ALT ratio (antagonistic association). This suggests that the gender-dependent strength of association for GGT and CRP levels and the AST-to-ALT ratio with MetS and its individual components underlines the protective effect of alcohol intake on MetS we found in our study.

MetS was associated with an increased risk of stroke-, either stroke or MI-, CVD-and all-cause death during the 9 year follow-up. After adjustment for age and other potential confounders, the risk was still more than 3 times higher for a fatal stroke and more than 2 times higher for death from either stroke or MI among men with MetS. The lack of significant associations between MetS and, CVD-, and, particularly, CHD-death in the adjusted analyses, might be due to heterogeneity of these diagnostic groups. The CHD, for example, included not only diagnoses of fatal myocardial infarction (I21-23) which are clinically well-distinguishable, characterized by progressing atherosclerosis and pathogenetically close to relatively homogenous and clinically well-defined group of cerebral strokes (I60-I64), but also such vaguely defined conditions as "other forms of acute or sub-acute ischemia" (I24) and "chronic ischemic heart disease" (I25). The latest evidences from Russia suggest that alcohol is an important factor implicated in the pathophysiology of the former two causes of death, and that some deaths within these subgroups are actually caused by acute alcohol intoxication[16] or alcoholic cardiomyopathy due to chronic toxic effects of alcohol on the myocardium[41,42]. We suggest that these CHD-subcategories should be included in future longitudinal analyses as separate end-points. However, it will require more statistical power which we lacked in the study. We also emphasize the need for larger population-based studies from Russia to either replicate or refute our results.

Thus, as a cluster of four major CVD risk predictors, MetS represents one of the factors contributing to the high cardiovascular mortality in Russia, but it is unlikely that it plays a central role at present. Following the improvements of living conditions during the last decade, the latest state's anti-alcohol initiatives launched in 2006, and the recent increase in life-expectancy [43], the prevalence of MetS is likely to increase in the nearest future, thereby enhancing the proportion of MetS-mediated cardiovascular and all-cause deaths in Russia.

## Conclusion

Age, GGT and C-reactive protein, and AST-to-ALT ratio were associated with MetS in both men and women. High leisure time physical activity in women and moderate frequency of alcohol consumption and binge drinking in men were inversely associated with MetS. Differences between men and women in alcohol consumption may explain gender variation in the MetS prevalence. MetS increased the risk of death from stroke and from either myocardial infarction or stroke during the 9-year follow-up period in men while no associations with mortality were found in women.

## Competing interests

The authors declare that they have no competing interests.

## Authors' contributions

OS and ON planned the study and were responsible for collection of data. OS and AG performed data analysis. OS drafted the manuscript, which was further elaborated by AG and ON. All authors read and approved the final version of the manuscript.

## Pre-publication history

The pre-publication history for this paper can be accessed here:

http://www.biomedcentral.com/1471-2458/10/582/prepub
